# Rhinocerebral Mucormycosis: A Ten-Year Single Centre Case Series

**DOI:** 10.7759/cureus.11776

**Published:** 2020-11-29

**Authors:** Edward Balai, Sangha Mummadi, Karan Jolly, Adnan Darr, Husham Aldeerawi

**Affiliations:** 1 Otolaryngology, The Royal Wolverhampton NHS Trust, Birmingham, GBR; 2 Otolaryngology, University Hospitals North Midlands, Stoke, GBR; 3 Otolaryngology, University of Basra Hospital, College of Medicine, Basra, IRQ

**Keywords:** rhinocerebral mucormycosis, invasive fungal disease

## Abstract

Introduction

Rhinocerebral mucormycosis (RCM) is a rare, frequently lethal, opportunistic infection of the paranasal sinuses and brain caused by fungi of the Mucoracea family. The overall global incidence is low, with the condition most commonly found in India and the Middle East. Early diagnosis and aggressive treatment are essential. Overall mortality is high; reported rates range from 25-60%. Its infrequent presentation can pose both diagnostic and therapeutic challenges for centers not familiar with the condition.

Objective

We aimed to evaluate patient demographics, clinical presentation, diagnosis, management, and the complications of this uncommon condition.

Methods

We carried out a retrospective case-series analysis of all patients with a confirmed diagnosis of RCM presenting to a single tertiary-level hospital between 2000-2010. Hospital patient records were used to attain the specific clinical details for each case.

Results

A total of nine patients (eight males and one female) were diagnosed with RCM during this period. All patients had diabetes mellitus; the mean age was 58.2 years. The most common presenting features were foul-smelling blood-stained rhinorrhoea (100%), nasal congestion (100%), reduced visual acuity (89%), and hard palate ulceration (67%). Two patients had a cerebral abscess at presentation; two patients had skull base erosions with associated cranial nerve palsies. All patients received systemic amphotericin B and surgical debridement. The overall mortality rate was 78%.

Conclusions

Rhinocerebral mucormycosis is a notoriously difficult infection to treat. Our case series demonstrates how patients often present late with a disease that has already spread beyond the paranasal sinuses. Despite treatment with antifungals and extensive surgical debridement, mortality remains high.

## Introduction

Mucormycosis is a rare, potentially fatal, opportunistic infection caused by fungi of the family Mucoraceae [1]. The disease can manifest as a variety of different syndromes, with the rhinocerebral and pulmonary infections being the most commonly reported [2]. The annual incidence of mucormycosis is estimated to range from 1.7 cases per 1,000,000 inhabitants in the United States of America to 140 cases per 1,000,000 in India and Pakistan [3]. While there are reports of the disease occurring in healthy individuals, it most commonly occurs in patients with diabetes mellitus or immunocompromised patients [2, 4].

Mucormycosis, and in particular rhinocerebral mucormycosis, carries high overall mortality [2]. It is a rare disease and therefore can pose diagnostic and therapeutic challenges for centers that are not familiar or experienced with its presentation. Early recognition and diagnosis followed by comprehensive surgical debridement, prolonged antifungal therapy, and tight control of underlying co-morbidities are essential [4]. We present a case series of nine patients diagnosed and treated for rhinocerebral mucormycosis at a single center. 

## Materials and methods

This was a retrospective case series analysis of all patients with a confirmed diagnosis of rhinocerebral mucormycosis presenting to a single tertiary-level hospital in Basra, Iraq, from 2000 to 2010. Patients were identified from history, examination, and a confirmed final diagnosis of rhinocerebral mucormycosis listed in departmental records. Hospital electronic records were used to attain the specific clinical details for each case. We evaluated patient demographics, clinical presentation, diagnosis, management, and complications of the disease. Statistical analysis was carried out using basic statistical functions in the Microsoft Excel program (Excel v. 16.0, Microsoft, Washington, USA). Written informed consent was obtained from each patient for their anonymized information to be published in this article. Ethics committee approval was not required for this study. Our institution does not require ethics committee approval for reporting individual cases or case series.

## Results

A total of nine patients (eight males and one female) were diagnosed with rhinocerebral mucormycosis during this 10-year period. The mean age at presentation was 58.2 years (range: 45-67 years). All nine patients had type-2 diabetes mellitus on insulin treatment regimes.

The patients presented with the following signs and symptoms: offensive blood-stained rhinorrhoea (100%), nasal congestion (100%), hard palate ulceration (67%), and cranial nerve palsy (22%). The ophthalmology department carried out formal eye assessment; 89% of patients were found to have reduced visual acuity, chemosis, and proptosis. Clinical findings for each patient are summarised in Table 1.

**Table 1:** Summary of patient demographic, clinical features and outcome

Demographics		Clinical features						Outcome
		Co-morbidity: type-2 diabetes mellitus	Visual/orbit: reduced visual acuity, chemosis, proptosis	Nasal: blood-stained discharge	Palatal: hard palate ulceration	Intracranial extension	Cranial nerve palsy	
1	50/M	Y	Y	Y	Y	N	N	Deceased
2	60/F	Y	Y	Y	N	Skull base erosion	Unilateral VII nerve	Long-term follow up
3	45/M	Y	Y	Y	Y	N	N	Long-term follow up
4	65/M	Y	Y	Y	Y	Cerebral abscess	N	Deceased
5	62/M	Y	Y	Y	N	N	N	Deceased
6	56/M	Y	Y	Y	N	N	N	Deceased
7	67/M	Y	Y	Y	Y	Skull base erosion	Unilateral VI + VII nerves	Deceased
8	53/M	Y	N	Y	Y	Cerebral abscess	N	Deceased
9	66/M	Y	Y	Y	Y	N	N	Deceased

Flexible nasal endoscopy and CT imaging of the paranasal sinuses were done in all cases to assess the extent of the disease. Documented endoscopic findings for all patients in the series were of dark nasal crusting, destruction of the turbinates and mucosa, and widening of the nasal cavity. Two patients were found to have cerebral abscesses involving the frontal and temporal lobes; two patients had skull base erosions that had led to cranial nerve palsies.

Histopathological analysis with Grocott-Gomori methenamine silver (GMS) fungal stain and culture in Sabouraud dextrose agar (SDA) were performed in all cases to confirm the diagnosis of rhinocerebral mucormycosis. All of the patients were treated with systemic amphotericin B at a dose of 3 mg/kg/day and endoscopic surgical debridement of the paranasal sinuses without orbital exenteration. One patient developed a cutaneous fistula after surgical drainage of a cerebral abscess in the frontal lobe. Despite medical and surgical treatment, seven out of the nine patients succumbed to the disease, giving an overall mortality rate of 78%. 

## Discussion

Rhinocerebral mucormycosis is an acute, angioinvasive fungal infection of the paranasal sinuses and brain. It is considered an opportunistic infection, typically developing in patients with impaired immune function due to poorly controlled diabetes mellitus, underlying malignancy, neutropenia, or the use of immunosuppressive agents [5]. Innate immunity involving macrophages and neutrophils is thought to be critical in the host defense against fungal infection, mediating hyphal damage through phagocytosis, oxidative and non-oxidative mechanisms [6]. Chemotherapy-induced neutropenia or steroid-induced immunosuppression of these processes, therefore increase the risk of an opportunistic fungal infection. In patients with diabetes, persistent hyperglycemia is thought to lead to a reduction in neutrophil phagocytosis and chemotaxis [7]. In addition to this, the acidic environment that accompanies diabetic ketoacidosis reduces the binding of iron to transferrin, increasing free iron concentration that promotes fungal multiplication [8].

The most common causative organisms are of the Rhizopus, Mucor, and Absidia species [1]. Infection of humans is thought to occur due to aerosolized fungal spores depositing on the mucosa of the nasal turbinates, with subsequent invasion and progression to involve the paranasal sinuses, orbits, and intracranial structures. Vascular invasion is a common feature; the fungus proliferates within the internal elastic lamina of the vessel, eventually breaching the endothelium to cause an infarction, hemorrhage, and subsequent tissue necrosis [9]. Diagnosis is confirmed through biopsy and histological analysis of the affected tissues. On microscopy, broad-based ribbon-like non-septate hyphae with irregular right-angled branching are the key diagnostic microscopic features [10]. Computed tomography imaging with the contrast of the paranasal sinuses and brain (if intracranial complications are suspected) should be carried out to determine the extent of the disease. Pathological findings include ethmoid or sphenoid sinusitis with orbital or intracranial extension, bony erosions, and cavernous sinus or internal carotid artery thrombosis [11].

Management of rhinocerebral mucormycosis requires surgical sino-nasal drainage and debridement of orbital or cerebral disease, combined with a prolonged course of intravenous antifungal medication. The limits of debridement will depend on the extent of the infection. In some cases, debridement of the paranasal sinuses and peri-orbital tissues will be enough. Whereas in others, it may need to extend back towards the skull base and cranium, requiring craniectomies, lobectomies, and orbital exenteration [12]. Serial debridement is often required to control the infection [4, 9]. First-line medical treatment consists of intravenous amphotericin-based antifungals, with a course of at least six weeks duration usually required. Liposomal formulations of amphotericin B, in particular, have proven efficacy and safety [13, 14]. If amphotericin-based treatment fails, second-line antifungals such as intravenous posaconazole can be trialed as salvage therapy [15, 16]. With the close association of the infection with diabetes mellitus and the state of diabetic ketoacidosis, strict metabolic control, and prevention of hyperglycemia is also a key aspect of treatment.

Regarding patient demographics, in-line with our report, previous case series have found the average age to be approximately 60 years [17, 18]. While the majority of the patients in our series were male, generally, it is reported to affect both genders with equal incidence. Diabetes mellitus is the most commonly reported predisposing factor for rhinocerebral mucormycosis. In our case series, all patients had type-2 diabetes established on insulin treatment. Similar case series by Nezafati et al. and Kolekar et al. reported the percentage of patients with underlying diabetes to be 90% and 80%, respectively [17, 18].

Clinical presentation can be diverse. The most commonly reported signs in the literature include blood-stained nasal discharge, facial swelling, palatal ulceration or necrosis, and black necrotic eschar in the nasal cavities [17, 18]. The most frequently reported ophthalmological signs are ophthalmoplegia, proptosis, loss of vision, and chemosis [19]. Intracranial infection may occur via spread within the vasculature or due to invasion through the cribriform plate [10]. Rhinocerebral mucormycosis frequently has a rapid and aggressive course, with palatal, orbital, and intracranial involvement developing over a matter of days [5]. In our case series, we found a similar pattern of presenting features; however, compared to other series, the vast majority of patients presented with signs of advanced disease that had already spread beyond the paranasal sinuses. Sixty-seven percent of patients in our series had palatal ulceration, whereas previous authors found this to be present in just 10-38% of patients [17, 18]. Almost half of our patients had developed an intracranial complication or cranial nerve palsy, whereas the previous series have reported this in just 10% [17].

As reported in the literature, mortality from rhinocerebral mucormycosis is high, ranging from 25% to 60% [5]. A successful outcome is dependent on early diagnosis and involvement of otolaryngology, ophthalmology, neurosurgical and medical teams. Late presentation, and thus the presence of orbital or intracranial complications, generally confers a poor prognosis in rhinocerebral mucormycosis [4]. In our case series, we encountered a relatively high percentage of patients presenting with advanced disease features, which is reflected in the high mortality rate of 78%. The healthcare system from where this patient series comes from is one that has a priority focus on hospital-based curative care and significantly less funding for the provision of primary care in the community [20]. An overloaded primary care service reduces a patient’s ability to both receive input into the management of a key risk factor like diabetes and to see a clinician early in an acute episode of disease [21].

## Conclusions

Rhinocerebral mucormycosis is an acute, invasive, and often fatal fungal disease. The condition most commonly affects those with underlying diabetes mellitus or immunosuppression. Diagnosis relies on histopathological analysis, with nasendoscopy and CT imaging performed to assess the extent of the disease. This case series demonstrates that while the overall incidence of this condition is very low, at an initial presentation, a patient may already have the extensive disease with orbital or intracranial complications. The mortality rate remains high, even despite aggressive treatment with systemic antifungal therapy and surgical debridement.
